# Impact of the BioFire FilmArray Meningitis‐Encephalitis Panel on Management of Suspected Paediatric Central Nervous System Infections: A Single‐Centre Retrospective Cohort Study

**DOI:** 10.1111/jpc.70303

**Published:** 2026-02-06

**Authors:** Louisa Mary Churcher, Adam William Bartlett, Stella Pendle, Rob Slade, Phoebe C. M. Williams

**Affiliations:** ^1^ Department of Paediatrics Northern Beaches Hospital Sydney New South Wales Australia; ^2^ School of Women and Children's Health University of New South Wales Sydney New South Wales Australia; ^3^ Department of Infectious Diseases Sydney Children's Hospital Randwick New South Wales Australia; ^4^ The Kirby Institute University of New South Wales Sydney New South Wales Australia; ^5^ Department of Microbiology Australian Clinical Labs Bella Vista New South Wales Australia; ^6^ Sydney Institute for Infectious Diseases, School of Public Health, Faculty of Medicine University of Sydney Sydney New South Wales Australia; ^7^ National Centre for Immunisation Research and Surveillance Sydney New South Wales Australia

**Keywords:** antimicrobials, BioFire, encephalitis, meningitis, PCR

## Abstract

**Background:**

Meningoencephalitis causes significant morbidity and mortality in children worldwide. Prompt diagnosis remains challenging, yet advances in molecular diagnostic technology have improved diagnostic performance. This study examined whether the introduction of the BioFire FilmArray meningitis/encephalitis (ME) multiplex panel impacted the diagnosis and management of children with suspected meningoencephalitis.

**Methods:**

We conducted a retrospective cohort study (January 2019 to July 2021) at a non‐tertiary hospital in Sydney, Australia, during which the BioFire ME panel became available. Patients < 16 years who had cerebrospinal fluid (CSF) sampling for investigation of meningoencephalitis were included. Demographic, clinical and microbiological data were extracted to evaluate time to infection diagnosis, antimicrobial rationalisation and duration, investigations undertaken and admission length.

**Results:**

There were 122 CSF samples collected from children with suspected meningoencephalitis; 70/122 (57.3%) before and 52/122 (42.7%) after BioFire ME panel introduction. Twenty‐eight (23.0%) children had BioFire ME testing. Seven bacterial and 27 viral microbiologically confirmed central nervous system (CNS) infections were identified. BioFire ME panel use was associated with faster median confirmation or exclusion of meningoencephalitis (12.6 vs. 48.0 h, *p* < 0.01, for bacterial and 12.6 vs. 71.5 h, *p* < 0.01, for viral causes), quicker pathogen identification in viral meningoencephalitis (8.1 vs. 48.7 h, *p* = 0.019) and earlier antimicrobial rationalisation (24.0 vs. 30.4 h, *p* < 0.01). Total antimicrobial duration, investigation numbers and hospitalisation duration were not reduced.

**Conclusions:**

The BioFire ME panel has the potential to reduce the duration of empiric broad‐spectrum antimicrobials and their associated adverse effects on children, as well as improve healthcare resource efficiencies.

## Brief Points

- What is already known on this topic:
  - There are challenges to diagnosing or excluding meningitis or encephalitis in septic children and neonates, including administration of antibiotics prior to CSF sampling, contamination of CSF samples with blood and the time taken to obtain directed PCR results.
  - Recent advances in molecular diagnostic technologies include syndromic panel‐based multiplex PCRs such as the BioFire Film‐Array Meningitis/Encephalitis panel, which has been shown to have high sensitivity and specificity.
  - Paediatric patients with potential central nervous system (CNS) infection are commonly treated with broad‐spectrum antimicrobials; prior studies in children and adults have shown variable results with regards to the BioFire ME panel's impact on antiviral and antibiotic prescribing and duration of stay.
- What this paper adds:
  - Use of the BioFire Meningitis‐Encephalitis panel led to quicker diagnosis or exclusion of paediatric CNS infections in a non‐tertiary metropolitan hospital setting reliant on off‐site microbiological testing.
  - There was an associated earlier rationalisation of antimicrobials, with potential positive implications for the microbiome, antimicrobial resistance and organ toxicity risk.
  - There was no demonstrable impact on overall intravenous (IV) antimicrobial therapy duration or the number of investigations undertaken, which may reflect clinician preference to continue to manage these patients cautiously until further evidence confirming the utility of multiplex PCR tests via larger, multicentre prospective studies is available.

## 1 Introduction

Meningitis and encephalitis are rare yet life‐threatening central nervous system (CNS) infections responsible for significant morbidity and mortality in children, particularly in the neonatal period [1]. Global estimates indicate 190 515 children < 5 years die from meningitis or encephalitis annually, including 22 636 neonates [2]. Differentiating meningoencephalitis from other clinical presentations is important when managing septic children; while awaiting diagnostic test results, patients are commonly empirically treated with broad‐spectrum antimicrobials. Exposure to these may have toxicity effects [3], alongside deleterious impacts on the microbiome [4], and potential selection and dissemination of antimicrobial resistance genes [5].

Diagnostic clouding can occur when cerebrospinal fluid (CSF) samples are obtained after empiric antibiotics, which rapidly impacts culture yield—the current ‘gold standard’ test for bacterial meningitis [6]. Other challenges include CSF contamination with blood, inability to exclude viral causes (even in the absence of CSF pleocytosis), and varying culture sensitivity between organisms [7]. Diagnostic uncertainty may prolong hospital admission and increase the financial, social and emotional burden of a child's hospitalisation.

Recently, there has been rapid advancement of molecular technologies for CNS infection diagnosis, including development of panels that simultaneously test for several common CNS pathogens [8]. The BioFire FilmArray Meningitis/Encephalitis (ME) multiplex polymerase chain reaction (PCR) panel (hereon referred to as the BioFire ME panel) allows rapid identification of 14 potential CSF pathogens, with high sensitivity (90%–92%) and specificity (97%–99%) reported [9, 10, 11], though this varies between targets [12]. The panel tests for six bacteria, seven viruses and one fungus (Table 1) [13].

**TABLE 1:** Pathogens targeted by the BioFire Meningitis‐Encephalitis panel.

Bacteria	Viruses	Fungi
*Escherichia coli* K1	Cytomegalovirus	*Cryptococcus neoformans/gattii*
*Haemophilus influenzae*	Enterovirus	
*Listeria monocytogenes*	Herpes simplex virus 1	
*Neisseria meningitidis*	Herpes simplex virus 2	
*Streptococcus agalactiae*	Human herpesvirus 6	
*Streptococcus pneumoniae*	Human parechovirus	
	Varicella zoster virus	

Previous studies evaluating BioFire ME panel use in children have revealed mixed findings, with some demonstrating reduced duration of empiric antiviral therapy [14, 15, 16, 17], but variable impact on length of antibiotic treatment and hospitalisation [15, 16, 17, 18, 19].

This study aimed to assess whether implementation of the BioFire ME panel altered management of children with suspected CNS infections at a non‐tertiary hospital reliant upon off‐site diagnostic testing. Primary outcome was to determine if BioFire ME introduction impacted duration and rationalisation of antimicrobial therapy. Secondary aims were to evaluate other impacts on the patient/hospital system, including time to diagnosis, numbers of investigations performed, duration of admission, requirement for definitive venous access, and inter‐hospital transfer, as well as concordance of the panel with directed PCRs.

## 2 Methods

### 2.1 Study Setting

The study was conducted at Northern Beaches Hospital, a 488‐bed metropolitan hospital in Sydney, Australia with 24 paediatric beds and a special care nursery admitting patients from 32 weeks' gestation. BioFire ME was introduced for suspected CNS infections (requiring Paediatric Infectious Diseases or Emergency Specialist approval) on 11th June 2020, at the off‐site microbiology laboratory performing cultures which was situated around 40 km away. This complemented routine viral/bacterial PCR testing, which remained available via another off‐site microbiology service a similar distance away.

### 2.2 Study Design

Children < 16 years with CSF sampled for suspected CNS infection between 1st January 2019 and 31st July 2021 were included, identified via microbiology databases at Australian Clinical Labs, the hospital's pathology provider. Electronic Medical Records (eMR) were reviewed to extract de‐identified data including: patient demographics, CSF timing and results, antimicrobial prescribing details (commencement, rationalisation, oral switch, cessation), time of diagnosis/exclusion of CNS infection, dexamethasone use, investigations undertaken, requirements for definitive venous access and inter‐hospital transfer, discharge time and re‐presentations. Where multiple CSF samples were obtained during a patient's admission, analysis was from the first sample.

### 2.3 Statistical Analysis

Data was analysed using SAS version 9.4 (copyright 2002–2012 SAS Institute Inc., Cary, NC, USA). Descriptive analysis and statistical tests of comparison were undertaken between the two time cohorts, and within the later cohort between those who were and were not tested with BioFire ME. Wilcoxon rank‐sum two‐sample tests were used to analyse differences between time and antimicrobial dosage variables. Hodges–Lehmann estimations were used to determine 95% confidence intervals (95% CI) for differences in medians. Time‐to‐event analyses, using the Kaplan–Meier method and log‐rank test, were performed for total antimicrobial duration and admission duration across cohorts, with death or inter‐hospital transfer considered censoring events. Chi‐square tests were performed to calculate odds ratios and relative risks to compare dexamethasone use, investigation numbers, requirement for definitive venous access or transfer and hospital re‐presentations.

## 3 Results

There were 122 children < 16 years with CSF samples collected for suspected CNS infection during the study period; 70 (57%) prior to BioFire ME panel availability (1st January 2019 to 11th June 2020), and 52 (43%) after (12th June 2020 to 31st July 2021), including 28/52 (54%) tested with BioFire (Figure S1). Median patient age was 34 days (IQR 13.0–80.5) with 56 neonates (46%), of whom 15/56 (27%) had not yet been discharged post‐birth and 41/56 (73%) were admitted from the community. Most patients were male (83/122, 68%). Demographic characteristics between groups were comparable (Table 2).

**TABLE 2:** Baseline demographics of included patients overall (Whole study) and comparing each time cohort before (Early cohort) and after (Late cohort) introduction of the BioFire ME panel, and of those in the Late cohort who did not (No BioFire ME) and did (BioFire ME) have a BioFire ME panel diagnostic test.

Demographic	Whole study (*n* = 122)	Early cohort (*n* = 70)	Late cohort		
			Whole cohort (*n* = 52)	No BioFire ME (*n* = 24)	BioFire ME (*n* = 28)
Median age (days) [IQR]	34 [13.0–80.5]	34 [10.5–77.3]	33 [13.8–100.5]	45 [13.0–1486]	31 [14.8–83.5]
Neonates	56/122 (46%)	33/70 (59%)	23/52 (41%)	11/24 (46%)	12/28 (43%)
Sex—female	39/122 (32%)	26/70 (37%)	13/52 (25%)	4/24 (17%)	9/28 (32%)
Sex—male	83/122 (68%)	44/70 (63%)	39/52 (75%)	20/24 (83%)	19/28 (68%)

### 3.1 CNS Pathogens Identified

**FIGURE 1:**
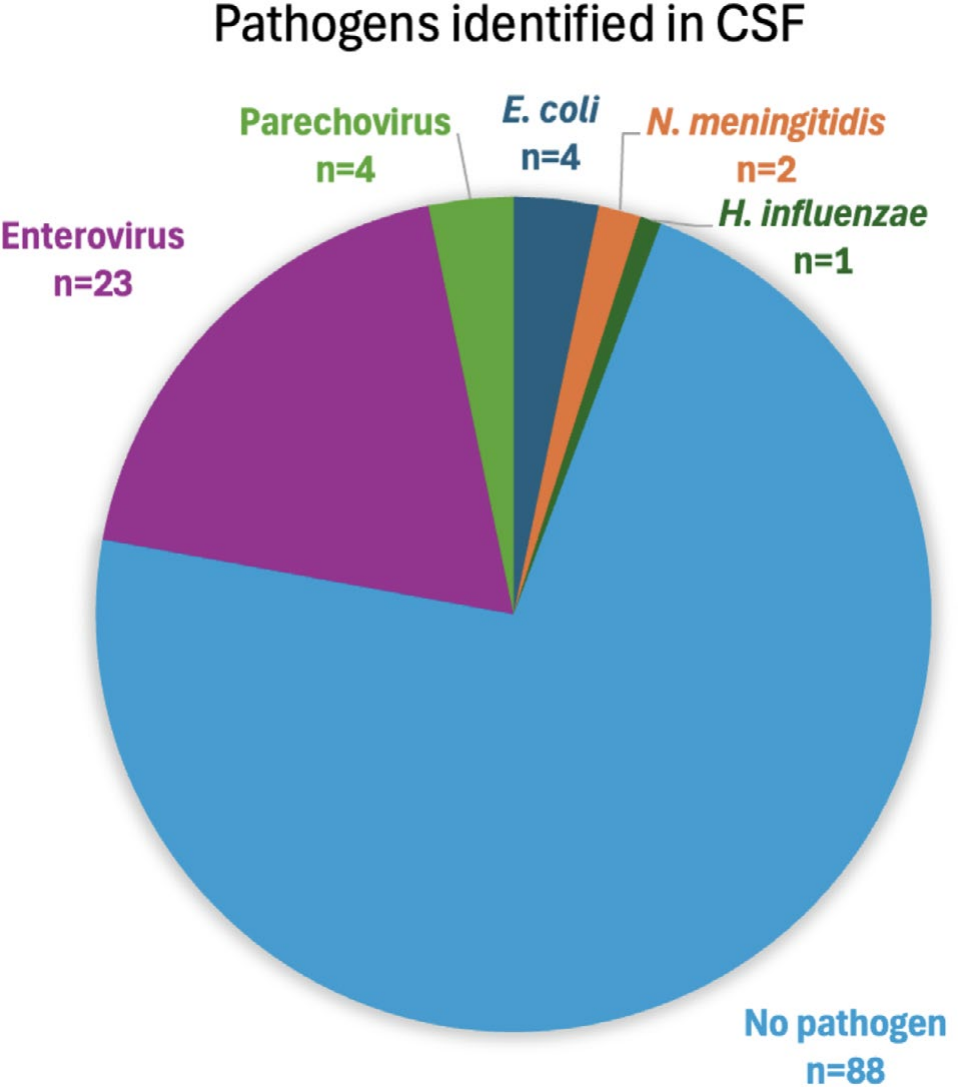
Pathogens identified in CSF by culture, directed PCR or BioFire ME panel as a percentage of total CSF samples taken for suspected CNS infection. *E. coli: Escherichia coli; H. influenzae: Haemophilus influenzae; N. meningitidis: Neisseria meningitidis*.

Of the 122 patients, 88 (72%) had no CSF pathogen identified. There were seven (6%) confirmed bacterial meningitis cases due to *Escherichia coli* [*n* = 4], *Neisseria meningitidis* [*n* = 2] and non‐typeable *Haemophilus influenzae* [*n* = 1]; six were culture‐positive, the seventh was obtained 4.7 h post‐ceftriaxone administration, with *N. meningitidis* detected by BioFire and directed PCR. All cultured organisms were susceptible to third‐generation cephalosporins; two of four *E. coli* cultures were resistant to ampicillin and gentamicin. There were 27 (22%) diagnosed viral CNS infections: enterovirus [*n* = 23] and parechovirus [*n* = 4] (Figure 1). Twelve pathogens were detected with BioFire ME ( *E. coli* [*n* = 3], enterovirus [*n* = 8] and *N. meningitidis* [*n* = 1]); the three *E. coli* detections were confirmed on culture, and the *N. meningitidis* and one enterovirus case had confirmatory directed PCRs (Table 3). Two patients had no pathogens detected on both BioFire ME and directed PCRs, giving complete concordance between the molecular tests [*n* = 4]. Overall, median time to positive CSF culture was 26.2 h (IQR 14.0–42.5), with susceptibility results after 54.9 h (IQR 42.1–68.2). Median time to directed PCR results was 61.9 h (IQR 47.2–96.4), versus 12.6 h (IQR 7.7–18.6) to BioFire ME results (*p* < 0.01).

**TABLE 3:** Positive pathogen detections by combination of diagnostic tests performed, before (Early cohort) and after (Late cohort) introduction of the BioFire ME panel.

Pathogen	Combination of diagnostic tests performed						Total
	Early cohort		Late cohort				
	Culture only (*n* = 8)	Culture + PCR (*n* = 62)	Culture only (*n* = 3)	Culture + PCR (*n* = 21)	Culture + BioFire (*n* = 24)	Culture + BioFire + PCR (*n* = 4)	
*E. coli*	1	—	—	—	3	—	4
*N. meningitidis*	—	1	—	—	—	1	2
*H. influenzae*	—	1	—	—	—	—	1
**Total positive for bacterial pathogen**	*1*	*2*	*0*	*0*	*3*	*1*	*7*
Enterovirus	N/A	12	N/A	3	7	1	23
Parechovirus	N/A	4	N/A	—	—	—	4
**Total positive for viral pathogen**	*N/A*	*16*	*N/A*	*3*	*7*	*1*	*27*

Abbreviations: *E. coli: Escherichia coli; N. meningitidis: Neisseria meningitidis; H. influenzae: Haemophilus influenzae*. N/A = not applicable (where bacterial culture alone could not detect viral pathogen).

### 3.2 Empiric Antimicrobials

**FIGURE 2:**
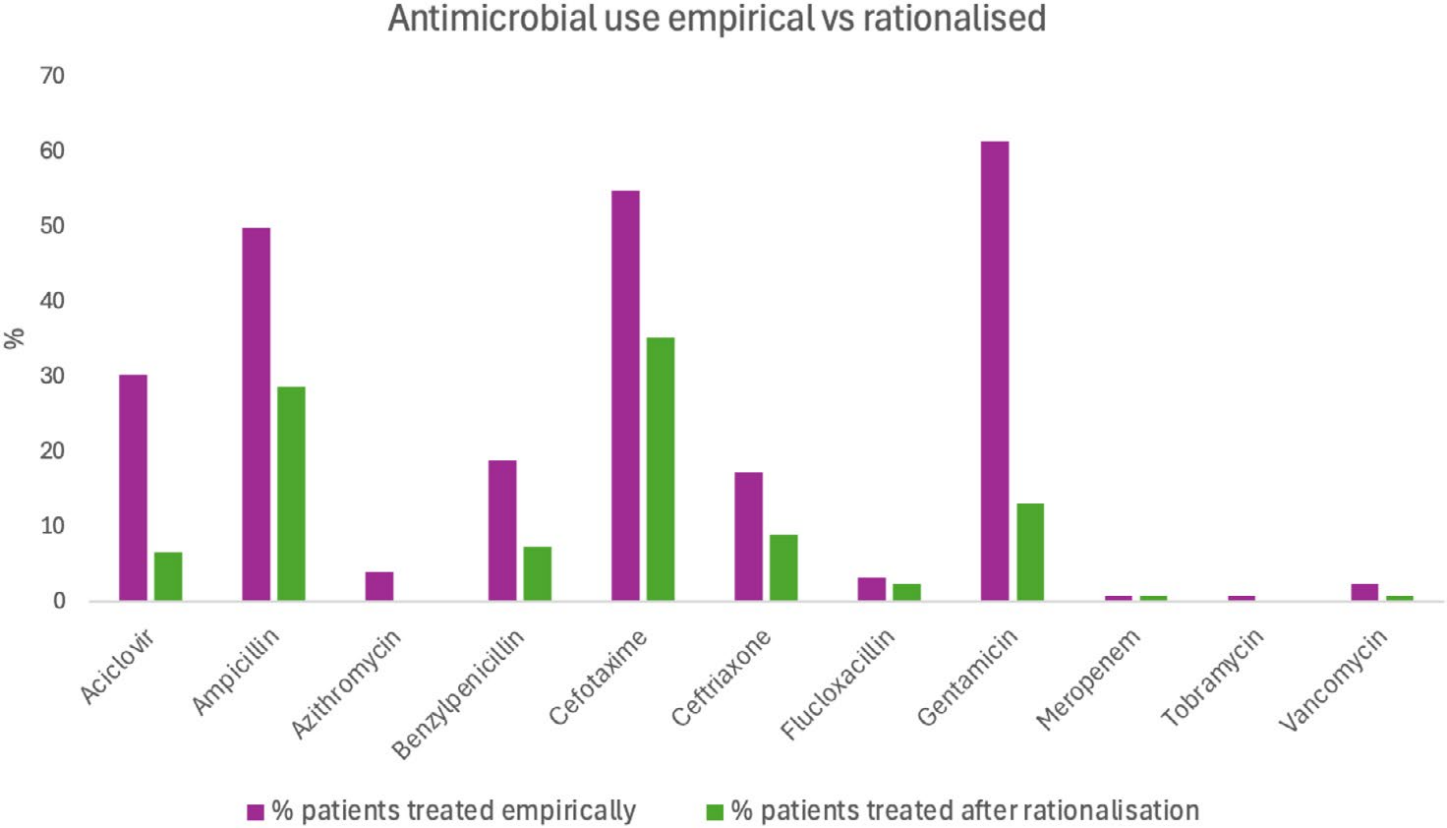
Percentage of all included patients receiving each antimicrobial empirically and after rationalisation to targeted therapy due to diagnostic test results.

Most patients were treated empirically with a third‐generation cephalosporin (cefotaxime/ceftriaxone) (88/122, 72.1%), gentamicin (75/122, 61.5%) and a penicillin‐based therapy (ampicillin/benzylpenicillin/flucloxacillin) (82/122, 67.2%), with 37/122 (30.3%) receiving aciclovir (Figure 2). At initial intravenous (IV) antimicrobial rationalisation/cessation, empiric aciclovir was ceased in 31/37 (83.8%), cephalosporins in 43/88 (48.9%) and penicillins in 39/82 (47.6%).

### 3.3 Comparison of Time Cohorts

Comparing cohorts before and after BioFire ME panel introduction, irrespective of diagnostic tool used, median time to confirmation/exclusion of bacterial meningitis (via culture or molecular methods) fell from 48.0 to 34.9 h (*p* < 0.01), alongside viral diagnosis/exclusion from 58.0 to 23.0 h (*p* < 0.01) and pathogen identification in CNS infection cases from 46.7 to 8.4 h (*p* < 0.01), including from 57.3 to 14.8 h (*p* < 0.01) in viral meningoencephalitis. Initial rationalisation/cessation of antimicrobials fell from 39.9 to 24.3 h overall (*p* < 0.01), and in viral meningoencephalitis from 34.6 to 19.9 h (*p* = 0.05). Total IV antimicrobial duration was not altered (from 59.0 to 52.0 h, *p* = 0.295), nor were total doses of IV antimicrobials (from 14.0 to 12.5, *p* = 0.189). However, for patients prescribed empiric aciclovir who had it ceased at first rationalisation, median time to this declined from 41.0 to 19.4 h (*p* < 0.01), alongside 39.8 to 24.5 h (*p* = 0.05) for empiric penicillins. Rationalisation of cefotaxime/ceftriaxone (from 48.0 to 30.0 h) was not significantly reduced (*p* = 0.47). Hospitalisation duration fell from 3.4 to 2.6 days (*p* = 0.034), but time‐to‐event analyses censoring at transfer showed no difference (log‐rank *p* > 0.05).

### 3.4 Comparison by Diagnostic Test

**FIGURE 3:**
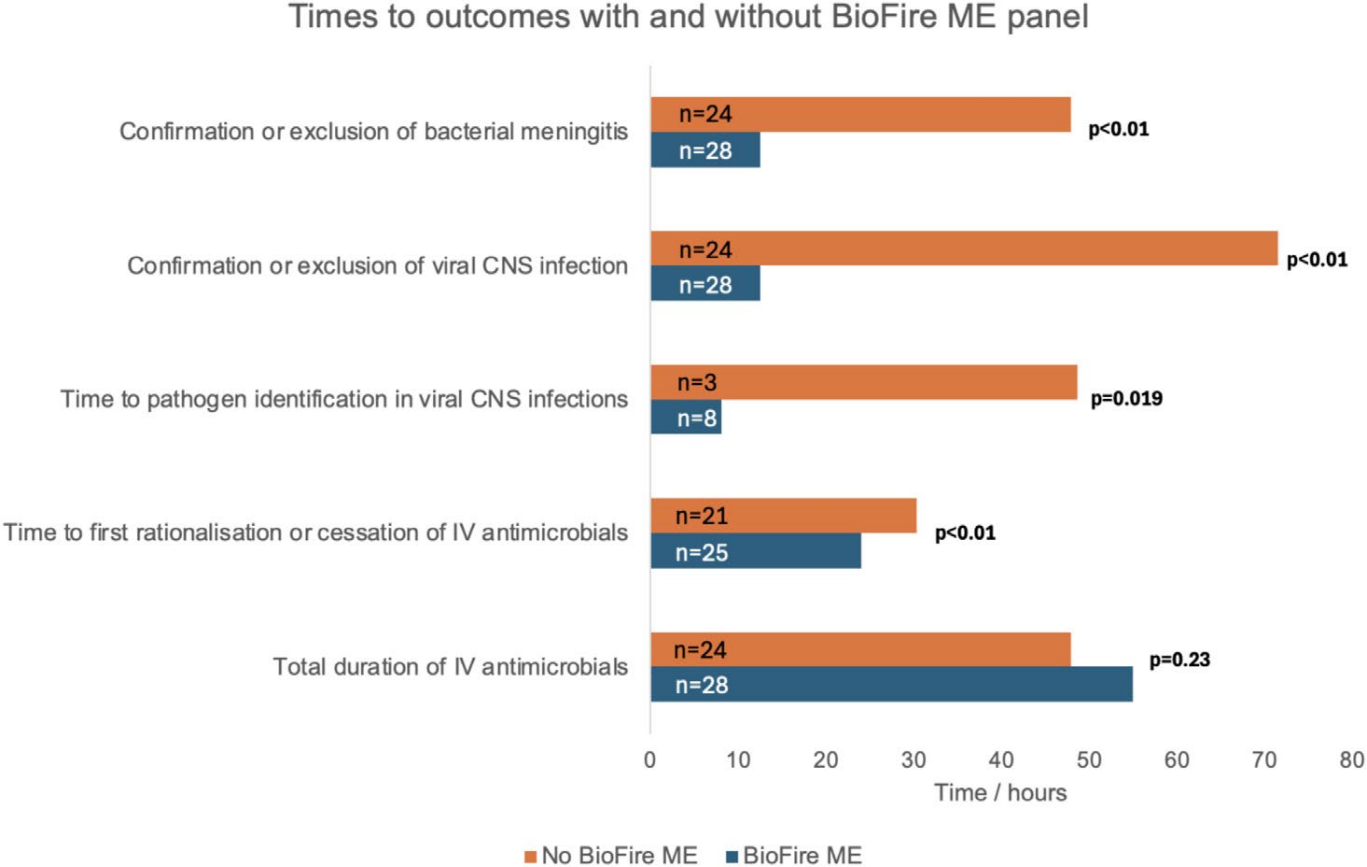
Median time in hours to confirmation or exclusion of bacterial and viral CNS infections, pathogen identification in viral CNS infection, first rationalisation or cessation of antimicrobials (excluding transferred patients) and total duration of IV antimicrobials, comparing the 24 patients in the later cohort who did not (No BioFire ME) and the 28 who did (BioFire ME) have a BioFire ME panel diagnostic test, including 11 cases of viral CNS infection. CNS: central nervous system.

Within the later cohort, time to bacterial meningitis diagnosis/exclusion was 12.6 h with BioFire ME performed versus 48.0 h without (*p* < 0.01), and for viral meningoencephalitis 12.6 versus 71.5 h (*p* < 0.01). All confirmed bacterial meningitis cases had BioFire ME; in the 11 viral meningoencephalitis cases diagnosis/pathogen identification was earlier at 8.1 versus 48.7 h (*p* = 0.019). With BioFire, empiric antimicrobials were first rationalised/ceased at median 24.0 versus 30.4 h (*p* < 0.01). Total IV antimicrobial duration was not reduced, at 55.0 versus 48.0 h (*p* = 0.23), (Figure 3); time‐to‐event analyses censoring patients at transfer also showed no difference (log‐rank *p* > 0.05). Total doses of IV antimicrobials (excluding transferred patients) were not reduced, at 13.0 versus 12.0 (*p* = 0.23).

**FIGURE 4:**
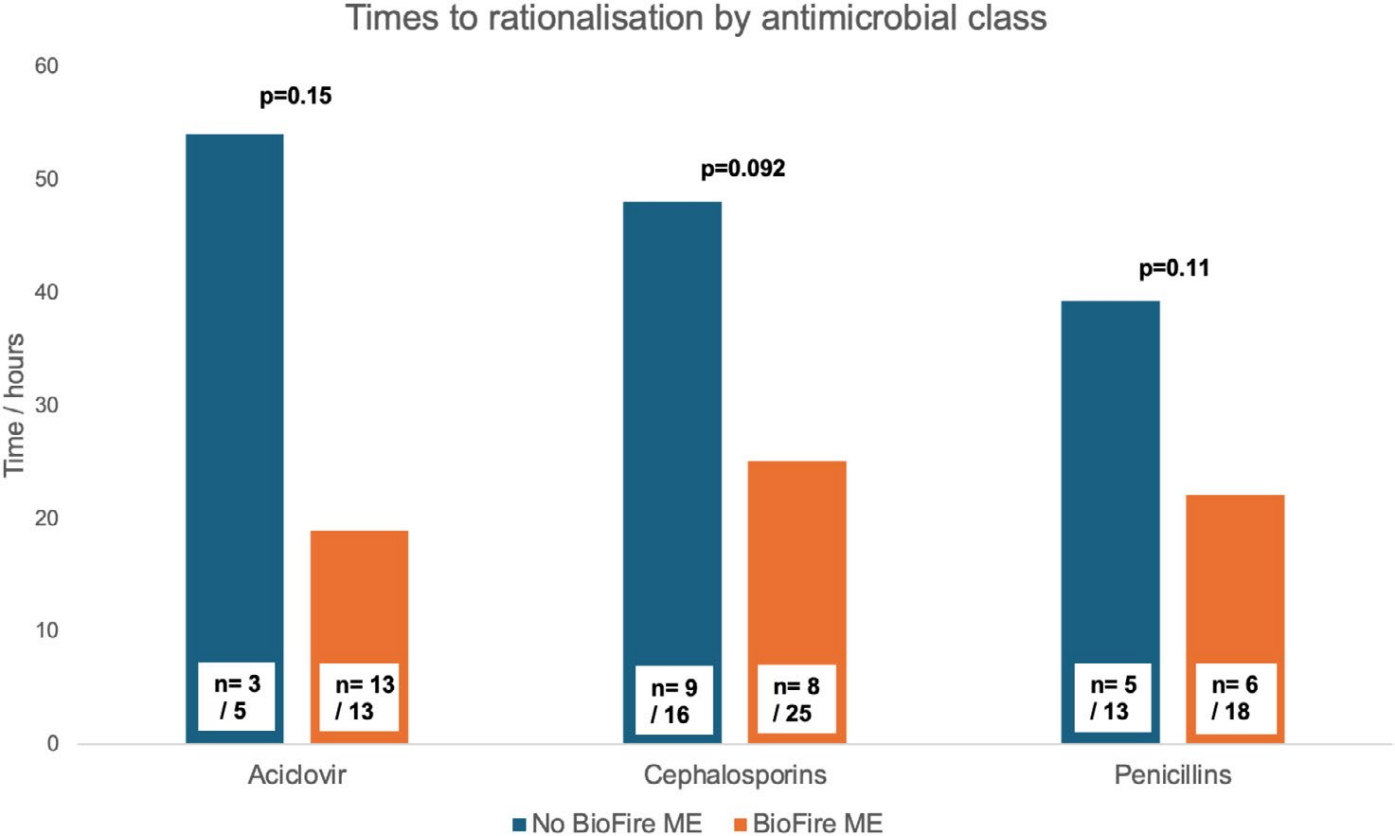
Time in hours to cease intravenous (IV) aciclovir, IV cephalosporins and IV penicillins in patients who had them commenced then ceased at first rationalisation (*n* = number rationalised/number commenced), comparing patients in the later cohort who did not (No BioFire ME) and did (BioFire ME) have a BioFire ME panel diagnostic test.

There were non‐significant trends towards reduction in median time to cessation at first antimicrobial rationalisation of empiric IV aciclovir (18.9 h with BioFire ME vs. 54.0 h without (*p* = 0.15)), cefotaxime/ceftriaxone (25.0 vs. 48.0 h (*p* = 0.092)) and penicillins (22.0 vs. 39.2 h (*p* = 0.11)) (Figure 4).

Use of BioFire ME was not associated with reduction in the number of other investigations, with similar numbers of blood tests (mean 2.6 (SD 2.3) vs. 1.8 (SD 1.0), *p* = 0.31), and brain imaging scans (11/28 (39.3%) vs. 9/24 (37.5%), (OR 1.08, 95% CI 0.35–3.31)) performed in both groups. Transfer for definitive intravenous access (RR 1.04, 95% CI 0.97–1.14), use of IV dexamethasone (OR 0.85, 95% CI 0.11–6.51) and midline/central venous catheter insertion (OR 2.76, 95% CI 0.27–28.5) were also similar between groups. Median hospital admission was longer with BioFire ME at 3.2 days versus 2.1 days (*p* = 0.014), including when censoring at transfer (log‐rank *p* = 0.021). There was no difference in re‐presentation to the hospital's Emergency Department within a week of discharge (0/28 vs. 1/24, RR 0.96, 95% CI 0.88–1.04) or 3 months (12/28 vs. 11/24, RR 0.95, 95% CI 0.58–1.54).

## 4 Discussion

Discerning meningoencephalitis from other febrile illnesses poses an ongoing diagnostic dilemma for clinicians caring for children. Challenges in diagnosing CNS infections and identifying causative pathogens are impacted by frequent empirical antimicrobial administration before diagnostic tests; this may be mitigated with sensitive rapid multiplex molecular assays like the BioFire ME panel [9, 10]. However, as most panels do not yet provide susceptibility data, they remain an adjunct to gold‐standard culture and susceptibility testing for bacterial pathogens.

Our study revealed that around one‐quarter of patients with suspected CNS infections had a pathogen identified, aligning with prior studies conducted on similar populations [19]. Over three‐quarters of CNS infections occurred in males, consistent with the known greater susceptibility and severity of infections in male children [20]. Aligning with other studies, the most commonly identified CSF pathogen was enterovirus, responsible for over two‐thirds of microbiologically confirmed CNS infections [16, 19].

Diagnostic results were obtained quicker with BioFire ME than directed PCRs and bacterial cultures, providing earlier diagnosis or exclusion of viral and bacterial CNS infections by well over a day. BioFire ME was associated with earlier first rationalisation/cessation of empirically‐prescribed antimicrobials by 6.4 h, and trends towards earlier cessation of aciclovir, cephalosporins and penicillins, though with insufficient sub‐group numbers to determine significance. Rationalisation of aciclovir and penicillins was significantly earlier once BioFire ME was available, but cannot definitively be attributed to it. Aciclovir carries a high risk of nephrotoxicity (especially when co‐administered with ceftriaxone) [21, 22], and cephalosporins' broad‐spectrum of activity may select antimicrobial resistance genes [23], detrimentally impact the microbiome, and cause organ toxicities [21, 24]. Prospective monitoring with a larger population could help determine harm reduction from avoided doses, as well as analyse resource efficiency.

We did not find a reduction in overall duration or doses of antimicrobials, nor length of hospitalisation, when the BioFire ME panel was utilised, aligning with some other studies [14, 16]. Earlier aciclovir rationalisation by 21.6 h once BioFire ME was available was more pronounced than in some similar studies [15, 16], but less than others [14]. Variable impact on antimicrobial prescribing may be influenced by study centre location and availability of other testing methods. One tertiary paediatric centre found no significant reduction in antibiotic duration with introduction of the panel [16], while a rural hospital found a significant decrease [19], and a systematic review and meta‐analysis combining adult and paediatric studies demonstrated non‐significant reduction of 1.01 days [14]. We found no significant impact on numbers of infection‐related blood tests, brain imaging, definitive line access or transfer requirement. In a population where investigations are rationed to minimise patient distress, a larger sample size might detect differences in these outcomes.

Limitations of the study include potential sampling bias, as BioFire ME use was not randomised. Whilst this promotes diagnostic stewardship, approval was liable to variability between clinicians and bias towards more vulnerable or unwell patients. Another limitation was variation in directed PCRs performed, a case‐by‐case clinical decision which ranged from single targets to a smaller multiplex panel targeting seven common pathogens ( *N. meningitidis* , *S. pneumoniae* , herpes simplex virus [HSV] 1 and 2, enterovirus, parechovirus and varicella zoster virus). Pathogens may have gone undetected, and time and cost to perform these ‘standard care’ tests might have varied depending on requests and with time. Though both labs were similar distances from the hospital, differing courier schedules and staffing could have impacted turnaround. Additionally, BioFire ME has been shown to vary in its sensitivity and specificity for different pathogens, in particular for HSV‐1 and 2, enterovirus and *Cryptococcus* [9], including a recent large study demonstrating only 82.4% sensitivity for HSV‐1 [12].

Although records were scrutinised for context, appropriateness of rationalisation decisions was not analysed and would have been influenced by non‐CNS pathological processes. Re‐presentations were captured from the hospital's eMR but would have missed presentations to other facilities. Furthermore, the COVID‐19 pandemic impacted the frequency of CSF sampling, with 53 in 2019, 29 in 2020 and 40 in the first 7 months of 2021. BioFire ME was introduced in the midst of the pandemic in June 2020, but overall incidence of meningoencephalitis was equivalent between time cohorts at an average of 1.1 cases/month, and viral cases comparable at 22.9% and 21.2%. The pandemic may have impacted admission lengths between time cohorts, with families and clinicians seeking earlier discharges.

The study's retrospective design and small numbers tested with BioFire ME rendered it under‐powered to detect differences in antimicrobial rationalisation between sub‐groups. A larger prospective study with randomisation or where all patients receive the panel might better elucidate its true potential impact on management, though universal application regardless of clinical need would bring increased costs. To our knowledge, broad implementation of the BioFire ME panel has not occurred in similar metropolitan settings to date, due to cost and because specialist input is required for accurate interpretation of results (such as delineating detection of potentially integrated non‐pathogenic HHV‐6 virus, and considering untested pathogens such as non‐K1 *E. coli)*. As most panels do not yet provide susceptibility data, they remain an adjunct to bacterial culture and susceptibility testing, and consideration must be given to pathogens not targeted. Future studies could evaluate the cost–benefit of earlier antimicrobial rationalisation, including accurate calculation of drug expenses, nursing and pharmacist time, number of cannula attempts, disposables and equipment (beyond the scope of this study), alongside the potential impact of reducing toxicities and microbiome alterations, to better quantify the benefits of BioFire ME use. Psychosocial benefits to patients and families of rapidly reaching or excluding a diagnosis are also important considerations.

## 5 Conclusions

In this non‐tertiary setting without onsite molecular testing, both availability and use of the BioFire ME panel led to quicker confirmation or exclusion of meningoencephalitis, faster organism identification and earlier antimicrobial rationalisation. The later cohort had earlier rationalisation of IV aciclovir and penicillins, but the difference was non‐significant comparing molecular test sub‐groups. Overall IV antimicrobial duration was not reduced, nor was investigation burden. Larger studies are required to prospectively define which patients would benefit from multiplex‐PCR‐based tests and monitor their implementation. The rapid, sensitive results afforded by new molecular diagnostic technologies can assist in narrowing broad‐spectrum antimicrobials and could reduce healthcare costs, with further evaluation required.

## Funding

The authors have nothing to report.

## Ethics Statement

The study was endorsed and approved to commence by the Northern Beaches Hospital QI/LNR Project Review Committee in December 2021 (project ID 2021/NBH/016) according to the National Statement on Ethical Conduct in Human Research. No Artificial Intelligence tools were used in the preparation of this manuscript.

## Conflicts of Interest

The authors declare no conflicts of interest.

## Supporting information


**Figure S1:**—Flow chart of patients meeting the inclusion criteria. CSF: cerebrospinal fluid; PCR: polymerase chain reaction.

## Data Availability

The data that support the findings of this study are available on request from the corresponding author. The data are not publicly available due to privacy or ethical restrictions.
