# Supplementary material for: Impact of the BioFire FilmArray Meningitis‐Encephalitis Panel on Management of Suspected Paediatric Central Nervous System Infections: A Single‐Centre Retrospective Cohort Study
Source: J Paediatr Child Health. 2026 Feb 6;62(4):586–93. doi: 10.1111/jpc.70303 (PMC13045770; doi:10.1111/jpc.70303)
Supplement: Supplementary file 1 — Figure S1:—Flow chart of patients meeting the inclusion criteria. CSF: cerebrospinal fluid; PCR: polymerase chain reaction. [file JPC-62-586-s001.docx]

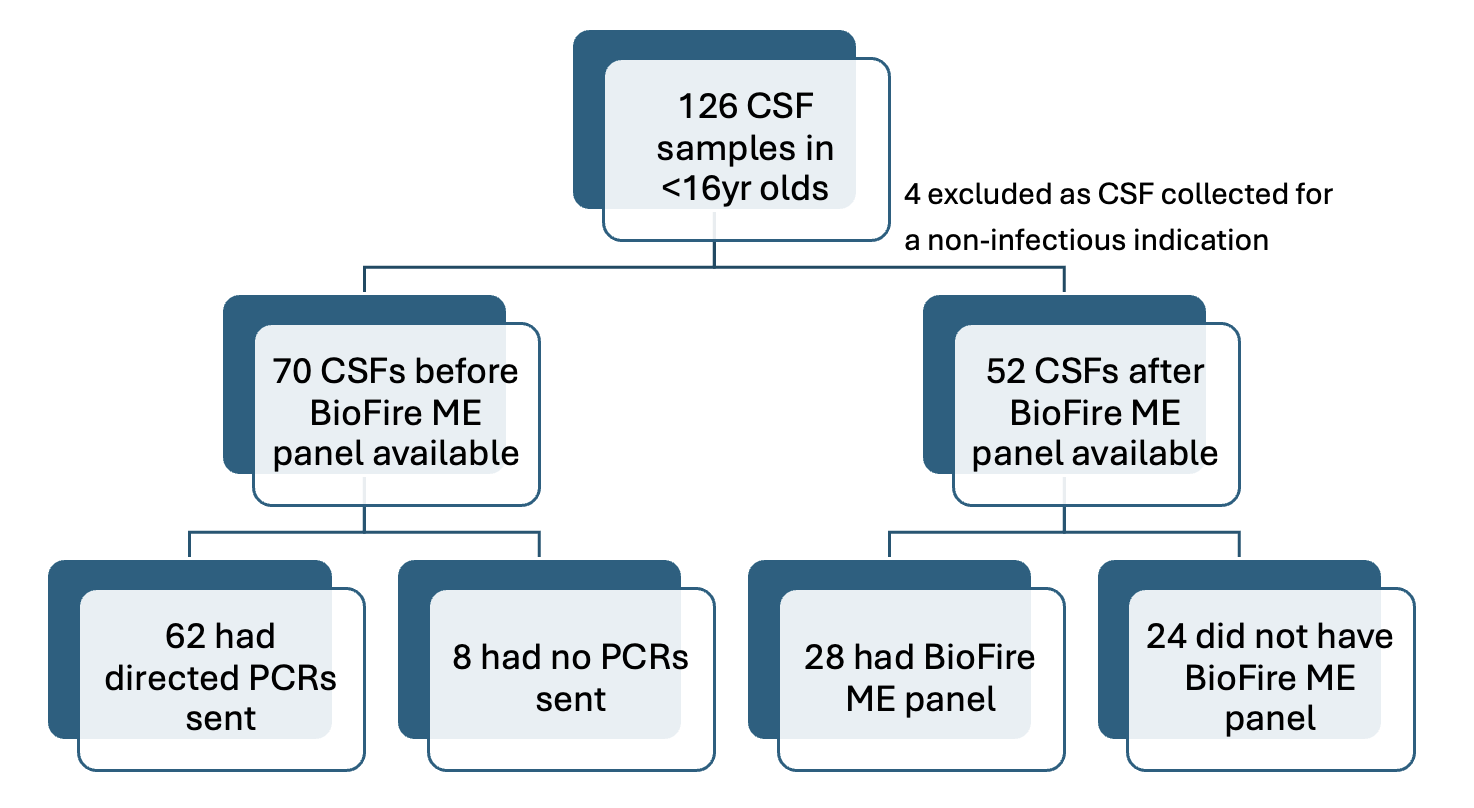


**Supplementary Figure 1** – Flow chart of patients meeting the inclusion criteria. CSF: cerebrospinal fluid; PCR: polymerase chain reaction.
